# Supplementary material for: A Toolkit for In Vivo Mapping and Modulating Neurotransmission at Single-Cell Resolution
Source: bioRxiv. 2025 Aug 18:2025.08.18.670838. Preprint. [Version 1] doi: 10.1101/2025.08.18.670838 (PMC12393400; doi:10.1101/2025.08.18.670838)

bioRxiv preprint doi: https://doi.org/10.1101/2025.08.18.670838; this version posted August 18, 2025. The copyright holder for this preprint (which was not certified by peer review) is the author/funder, who has granted bioRxiv a license to display the preprint in perpetuity. It is made available under aCC-BY 4.0 International license.

Figure S1

(A) Graphical representation of conservation of the amino acid predicted structure for the Vesicular Glutamate Transporter across common model organisms (named in Figure S1B). Dark colors denote the least conserved region, while clear colors denote the highest conservation. Note the C-terminus end, where we introduced the GFP, is one of the darkest (least conserved) regions in the structure. **(B)** Sequence alignment of the amino acid sequences for the Vesicular Glutamate Transporter across common model organisms. Blue letters indicate conservation of the amino acid properties (basic, acid, neutral) but not identities. Gray represents columns with gaps and no conservation.

(C) Schematic of chemotaxis assay. (Top) A concentration gradient of NaCl (~60-85 mM) is established on assay plates (blue rim) by adding high concentration NaCl drops to the outer point of the assay plate at pre-determined times (See Methods). (Bottom) Larva stage 4 animals are picked, and twenty-four hours later, the young adult animals are transferred to NaCl 100mM training plates (red rim) for five hours. Animals are transferred to NGM buffer drops to wash off bacteria, before being placed on the assay plates (blue rim, placement at blue dot in schematic), where animal movement away from the highest [NaCl] is recorded (towards red dot in schematic).

(D) Displacement of trained wild type animals on a sham gradient (13.92 ± 5.3 mm) or untrained wild-type animals on a gradient of NaCl (13.84 ± 5.4 mm). Wild-type animals (7.76 ± 5.2 mm) migrate across the salt gradient like EAT-4::GFP FLP-on animals that express (8.36 ± 5.8 mm) flippase in ASE neurons. Animals that only express flippase in ASE neurons (7.61 ± 5.3 mm) migrate across the salt gradient similar to wild-type animals. Results represent the mean distance of each worm from the salt peak, averaged across the final minute of the assay, with each dot representing an individual animal. Plots are overlaid with Mean ± Standard Deviation. Kruskal-Wallis test with Dunn's multiple comparison post hoc test. **** represents p<0.0001; *** represents p<0.001; * represents p<0.05; and NS means "not significant".

bioRxiv preprint doi: https://doi.org/10.1101/2025.08.18.670838; this version posted August 18, 2025. The copyright holder for this preprint (which was not certified by peer review) is the author/funder, who has granted bioRxiv a license to display the preprint in perpetuity. It is made available under aCC-BY 4.0 International license.

Figure S2

(A) Graphical representation of conservation of the Vesicular GABA Transporter amino acid predicted structure across common model organisms (named in Figure S2B). Dark colors denote the least conserved region, while clear colors denote the highest conservation. **(B)** Sequence alignment of the cytosolic loop between transmembrane domains 2 and 3 of the vesicular GABA transporter across common model organisms. Red letters indicate highly conserved columns

bioRxiv preprint doi: https://doi.org/10.1101/2025.08.18.670838; this version posted August 18, 2025. The copyright holder for this preprint (which was not certified by peer review) is the author/funder, who has granted bioRxiv a license to display the preprint in perpetuity. It is made available under aCC-BY 4.0 International license.

(conservation of amino acid identity), and blue letters indicate conservation of the amino acid properties (basic, acid, neutral) but not identities. Gray represents columns with gaps. **(C)** Schematic of thrashing assay. “One thrash cycle” is scored when the animal bends as indicated in the schematic (from elongated-to-bent-to-elongated). We measured the number of thrashes per minute. **(D)** *unc-47(e307)* mutant animals thrash significantly less (41.5 ± 21.35) than wild-type animals (109.2 ± 23), while over-expression of UNC-47::GFP (100.2 ± 16), CRISPR knock-in UNC-47::GFP (107.9 ± 29) and UNC-47::mKate2 (103.1 ± 18) CRISPR-tagged animals thrash similarly to wild-type animals. Plots are overlaid with Mean ± Standard Deviation. Kruskal-Wallis test with Dunn’s multiple comparison post hoc test. **** represents p<0.0001; *** represents p<0.001; ** represents p<0.01; * represents p<0.05; and NS means “not significant”. **(E-F)** Fluorescent image of endogenously tagged **(E)** UNC-47::GFP (*syb6990*) and **(F)** UNC-47::mKate2 (*syb7358*). DNC = Dorsal Nerve Cord, VNC = Ventral Nerve Cord. Scale Bar = 10μm.

Figure S3

(A) All presynaptic sites from known GABAergic neurons in the *C. elegans* nerve ring (RIS, RME, and RIB), according to 3D electron microscopy of a Larva stage 4 (L4) wild-type animal (White et al., 1986) (image generated with NeuroSC (Koonce et al., 2025)). **(B)** 3D-rendering of UNC-47::GFP (green) puncta in the *C. elegans* nerve ring. A-P denotes anterior-posterior axis. Scale bar = 10 μm. **(C)** Addition of RIB::BFP into Figure S3B. Green arrowheads point to UNC-47::GFP puncta that overlap with RIB::BFP. Yellow arrowheads point to the synapses we interpret, based on our labeling and distribution, to belong to the RME neurons. **(D)** *In-vivo* reconstitution of UNC-47::GFP11x3 with an RIB-specific GFP1-10 construct. Scale bar = 10 μm. Note that the only synapses detected come from RIB and not the other GABAergic cells in the nerve ring (compare toFigure S3B). **(E)** *In-vivo* reconstitution of UNC-47::GFP11x3 in DD neurons when tagged with (Top) one or (Bottom) three copies of GFP11 (P*flp-13*::GFP1-10) (He et al., 2019). **(F-G)** **(F)** Line scans and **(G)** quantification of reconstituted GFP fluorescence intensity with one (11.3 ± 6) or three copies (38.3 ± 28) of GFP11 in DD neurons. Scale bar = 10 μm. Plots are overlaid with Mean ± Standard Deviation. Mann-Whitney test. **** represents p<0.0001.

bioRxiv preprint doi: https://doi.org/10.1101/2025.08.18.670838; this version posted August 18, 2025. The copyright holder for this preprint (which was not certified by peer review) is the author/funder, who has granted bioRxiv a license to display the preprint in perpetuity. It is made available under aCC-BY 4.0 International license.

Figure S4

(A) Graphical representation of the amino acid conservation of the Vesicular Acetylcholine Transporter (VAChT) across common model organisms. Dark colors denote the least conserved region, while clear colors denote the highest conservation. Note the C-terminus end is one of the darkest regions in the structure, where the fluorophore was added. **(B-C)** Amino acid sequence alignment of VAChT across model organisms. Black arrowhead points to insertion site of fluorescent tag. **(B)** Sequences between transmembrane domains 6 and 7, which was tagged in *olaEx5704* array (See panel E). **(C)** Sequence at the C-terminus end. Red letters indicate highly conserved columns (exact conservation of the amino acid identity), and blue indicates conservation of the amino acid properties (basic, acid, neutral) but not identities. Gray represents areas of no conservation. **(D)** Fluorescence image of CRISPR-tagged UNC-17::mKate2 (*ot907*) animal. Scale Bar = 10μm. **(E)** Schematic of UNC-17 predicted topology (magenta) along the membrane (gray). Green represents the locations of fluorescent tags tested for thrashing assays. Alleles, either extrachromosomal arrays expressed in *unc-17* (*e245*) mutants or CRISPR knock-

bioRxiv preprint doi: https://doi.org/10.1101/2025.08.18.670838; this version posted August 18, 2025. The copyright holder for this preprint (which was not certified by peer review) is the author/funder, who has granted bioRxiv a license to display the preprint in perpetuity. It is made available under aCC-BY 4.0 International license.

in strains in the *unc-17* locus, are listed based on where the fluorescent tag was inserted. **(F)** *unc-17(e245)* mutant animals barely swim (1.7 ± 2). Over-expression of untagged P*unc-17*::UNC-17 (*olaEx5703*) (114.8 ± 18) and N-terminus-tagged UNC-17::GFP (*olaEx5400*) (91.1 ± 22) swim like wild-type animals (99.4 ± 20). GFP-tag between TM6-7 (*olaEx5704*) (34.3 ± 19) or CRISPR-insertion of C-terminus tag (*ot907*) (77.7 ± 17) leads to reduced swimming behavior when compared to wild-type animals. Insertion of GFP-FLP-on cassette at the N-terminus end (s*yb7251*) (82.9 ± 16) or insertion of the GFP-FLP-on cassette at the C-terminus (*ola503*) (101.9 ± 16) thrash as well as wild-type animals (109 ± 25). Mean ± Standard Deviation. Kruskal-Wallis test with Dunn's multiple comparison post hoc test. **** represents $p<0.0001$; ** represents $p<0.01$; * represents $p<0.05$; and NS means "not significant".

bioRxiv preprint doi: https://doi.org/10.1101/2025.08.18.670838; this version posted August 18, 2025. The copyright holder for this preprint (which was not certified by peer review) is the author/funder, who has granted bioRxiv a license to display the preprint in perpetuity. It is made available under aCC-BY 4.0 International license.

Figure S5

(A) To track glutamate-involved co-transmission, we repurposed the *eat-4* conditional KO strain (*kySi76 kySi77*) (Lopez-Cruz et al., 2019) where the *eat-4* gene coding sequence is flanked by two FRT sites and followed by cytosolic mCherry (Element #2, orange box). Crossing this line with a panel of flippase drivers (Element #1, blue box) results in activation of cytosolic mCherry only in cells were both elements were co-expressed (Readout, green box). P*dat-1*::Flippase and P*tph-1*::Flippase driver strains were made available from (Muñoz-Jiménez et al., 2017). **(B)** To track acetylcholine-involved co-transmission, we repurposed the unc-17::GFP conditional KI strain (*ola503*) (Figure 3B) for which, after the *unc-17* gene coding sequence, there are FRT sites

bioRxiv preprint doi: https://doi.org/10.1101/2025.08.18.670838; this version posted August 18, 2025. The copyright holder for this preprint (which was not certified by peer review) is the author/funder, who has granted bioRxiv a license to display the preprint in perpetuity. It is made available under aCC-BY 4.0 International license.

Figure S6

Live-imaging of co-expression strategy of the *eat-4* conditional knockout strain (*kySi76 kySi77*) with **(A)** endogenous P*unc-47*::UNC-47::T2A::Flippase and **(B)** P*dat-1*:: Flippase driver. Live-imaging of co-expression strategy of the *unc-17* conditional GFP knock-in strain (*kySi76 kySi77*) with **(C)** P*tph-1*:: Flippase driver, and with **(D)** endogenous P*unc-47*::UNC-47::T2A::Flippase. (Top) DIC images. (Bottom) Fluorescence imaging. All scale bars = 10μm.

Figure S7

Our results are consistent with previous studies in the field (Taylor et al., 2021; Wang et al., 2024) and help establish an atlas of co-transmission in the *C. elegans* nervous system. Co-transmitter neurons are present in the (i) head, mid-body region and (ii) tail of the animal.

bioRxiv preprint doi: https://doi.org/10.1101/2025.08.18.670838; this version posted August 18, 2025. The copyright holder for this preprint (which was not certified by peer review) is the author/funder, who has granted bioRxiv a license to display the preprint in perpetuity. It is made available under aCC-BY 4.0 International license.

Figure S8

(A) Schematic of genes required for serotonergic identity. Mammalian (bold) and *C. elegans* (italics) homologue genes are listed. Detectable expression of **(A')** *tph-1,* **(A'')** *bas-1* and **(A''')** *mod-5* in ADF neuron (blue). Scale Bar = 5 μm**.**

(B) Schematic of genes required for cholinergic identity. Mammalian (bold) and *C. elegans* (italics) homologue genes are listed. Detectable expression of **(B')** *cho-1* in ADF neuron (blue). Scale Bar = 10 μm**.**

(C) (Left) Presynaptic densities (green) on ADF axons as determined by serial electron microscopy reconstructions (White et al., 1986) (image generated with NeuroSC (Koonce et al., 2025). (Right) Live-imaging of endogenous UNC-17::GFP and CAT-1::GFP specifically in ADF neurons.

Figure S1

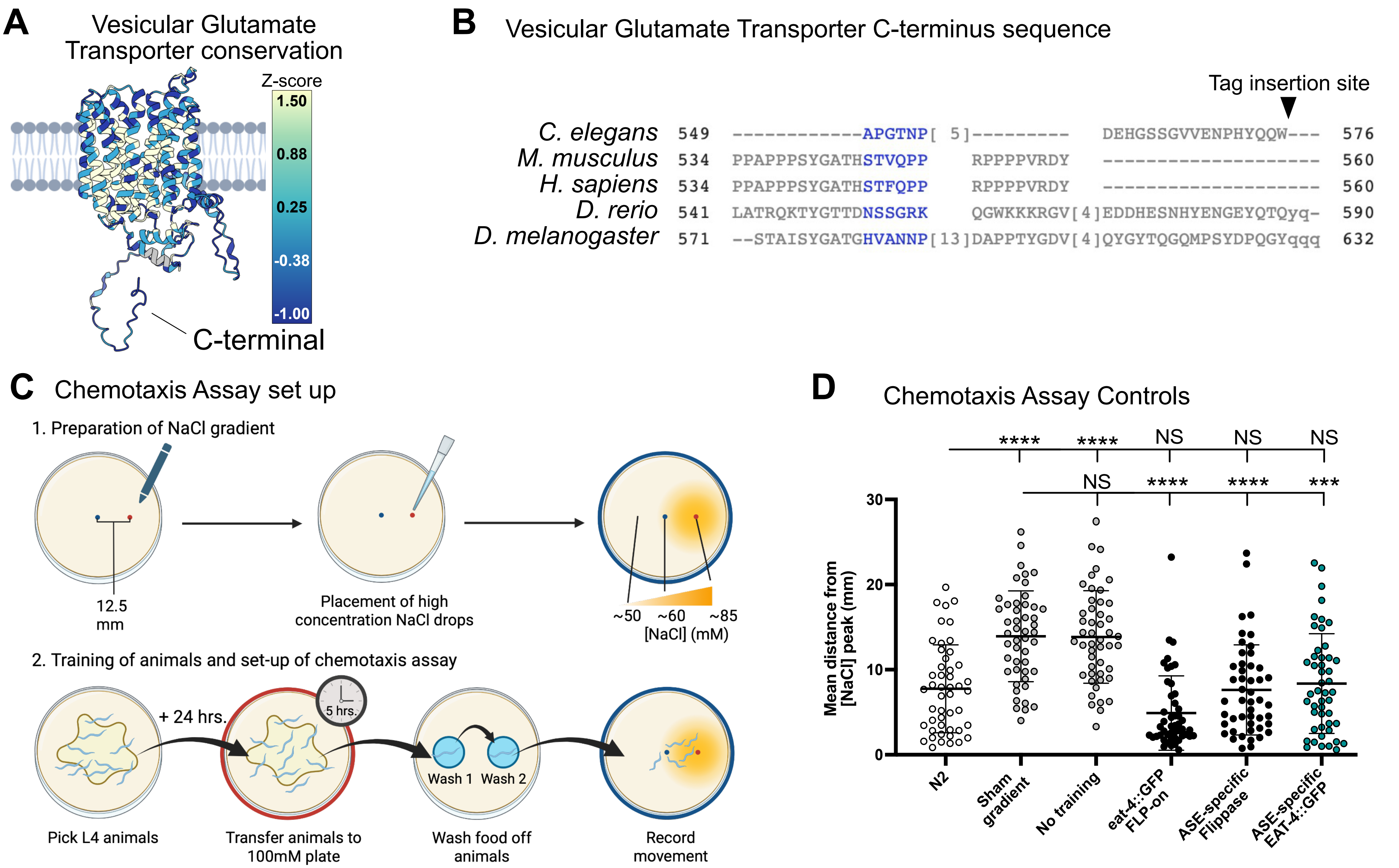

A
Vesicular Glutamate Transporter conservation
Z-score
1.50
0.88
0.25
-0.38
-1.00
C-terminal
B
Vesicular Glutamate Transporter C-terminus sequence
Tag insertion site
C. elegans 549 ------------APGTNP[5]--------- DEHGSSGVVENPHYQQW--- 576
M. musculus 534 PPAPPPSYGATHSTVQPP RPPPPVRDY -------------------- 560
H. sapiens 534 PPAPPPSYGATHSTFQPP RPPPPVRDY -------------------- 560
D. rerio 541 LATRQKTYGTTDNSSGRK QGWKKKRGV[4]EDDHESNHYENGEYQTQyq- 590
D. melanogaster 571 --STAISYGATGHVANNP[13]DAPPTYGDV[4]QYGYTQGQMPSYDPQGYqqq 632
C
Chemotaxis Assay set up
1. Preparation of NaCl gradient
12.5 mm
Placement of high concentration NaCl drops
~50 ~60 ~85
[NaCl] (mM)
2. Training of animals and set-up of chemotaxis assay
+ 24 hrs.
5 hrs.
Wash 1
Wash 2
Pick L4 animals
Transfer animals to 100mM plate
Wash food off animals
Record movement
D
Chemotaxis Assay Controls
**** **** NS NS NS
NS **** **** ***
Mean distance from [NaCl] peak (mm)
30
20
10
0
N2
Sham gradient
No training
eat-4::GFP FLP-on
ASE-specific Flippase
ASE-specific EAT-4::GFP

Figure S2

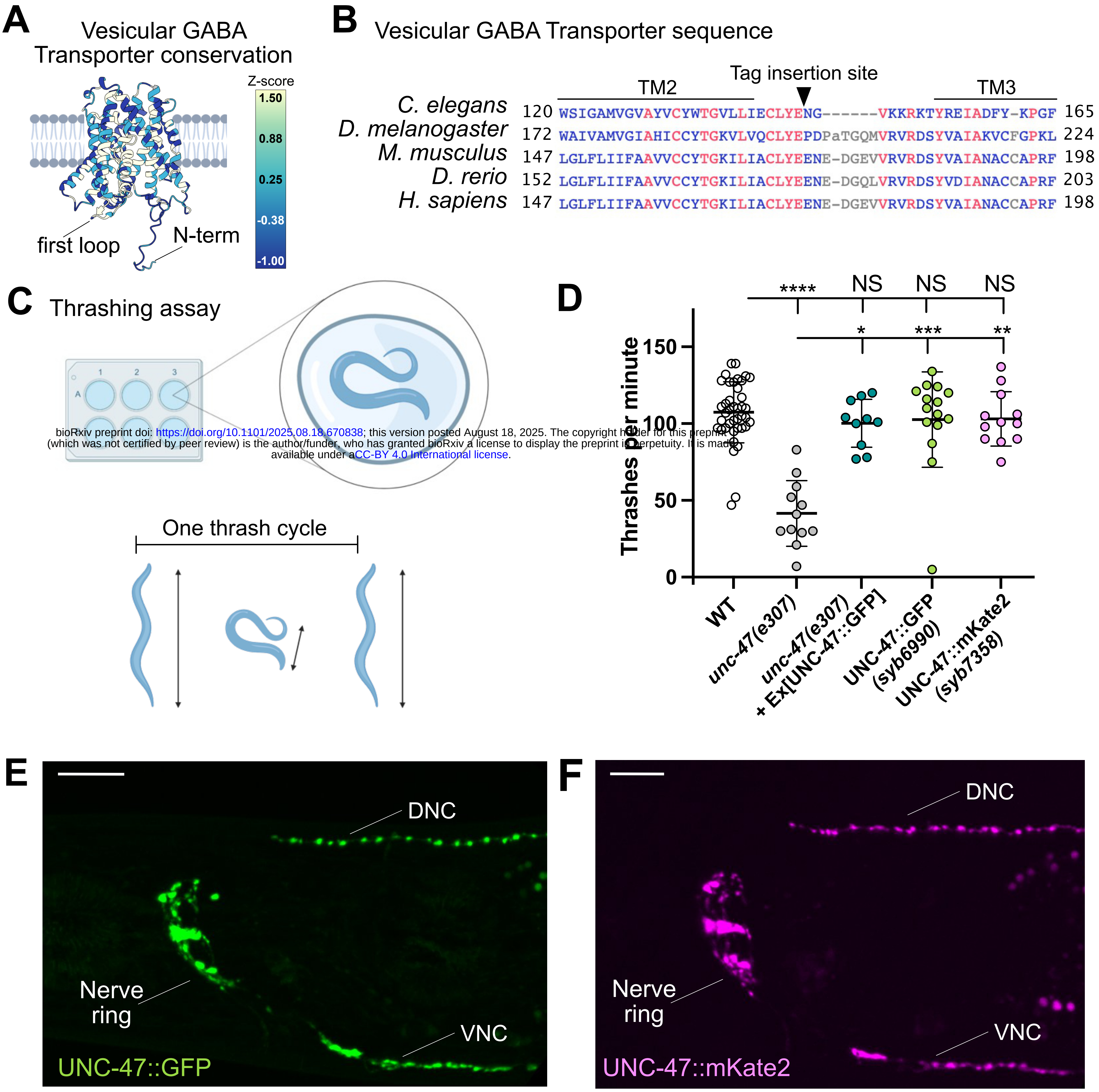

bioRxiv preprint doi: https://doi.org/10.1101/2025.08.18.670838; this version posted August 18, 2025. The copyright holder for this preprint (which was not certified by peer review) is the author/funder, who has granted bioRxiv a license to display the preprint in perpetuity. It is made available under aCC-BY 4.0 International license.

Figure S3

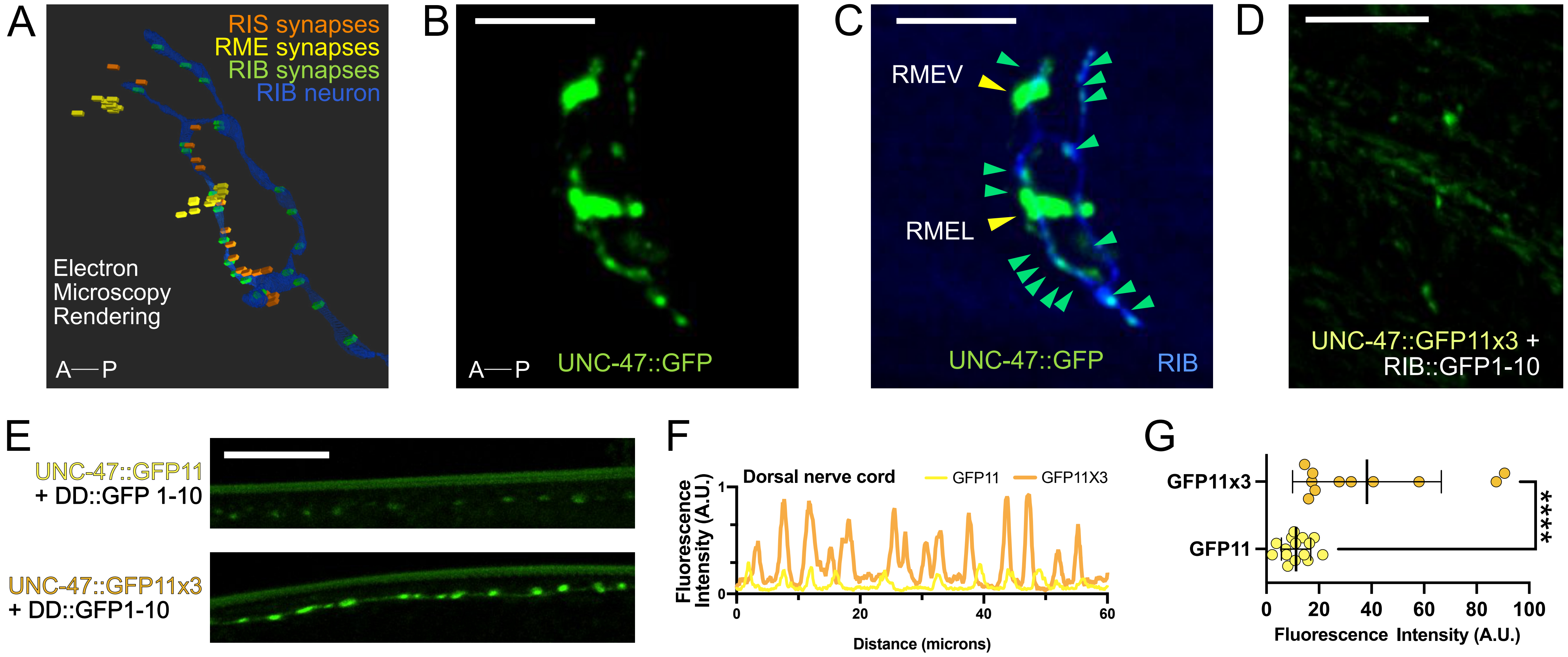

A
RIS synapses
RME synapses
RIB synapses
RIB neuron
Electron Microscopy Rendering
A—P
B
A—P
UNC-47::GFP
C
RMEV
RMEL
UNC-47::GFP
RIB
D
UNC-47::GFP11x3 +
RIB::GFP1-10
E
UNC-47::GFP11
+ DD::GFP 1-10
UNC-47::GFP11x3
+ DD::GFP1-10
F
Dorsal nerve cord
GFP11
GFP11X3
Fluorescence Intensity (A.U.)
1
0
0
20
40
60
Distance (microns)
G
GFP11x3
GFP11
****
0
20
40
60
80
100
Fluorescence Intensity (A.U.)

Figure S4

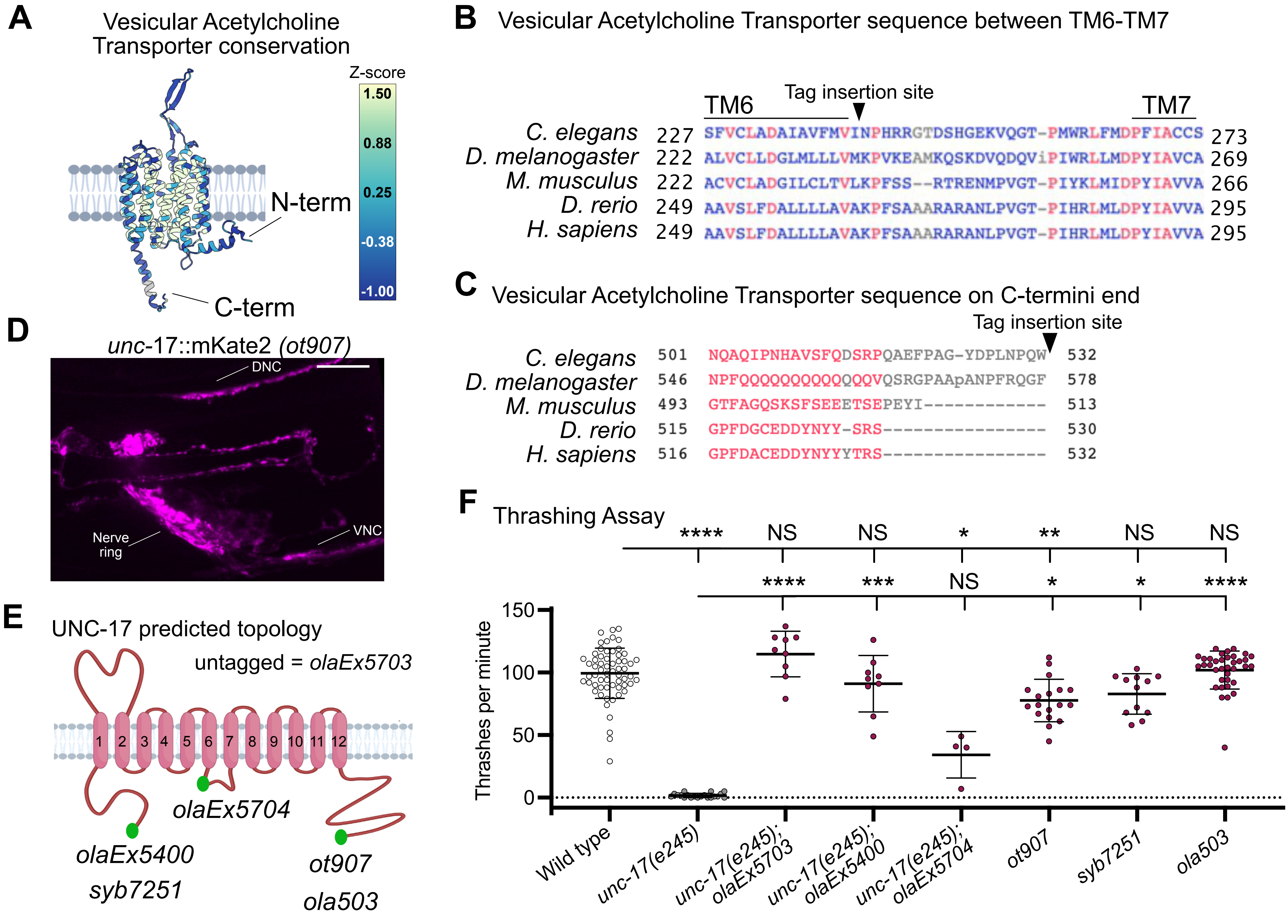
A
Vesicular Acetylcholine Transporter conservation
Z-score
1.50
0.88
0.25
-0.38
-1.00
N-term
C-term
B
Vesicular Acetylcholine Transporter sequence between TM6-TM7
TM6
Tag insertion site
TM7
C. elegans 227 SFVCLADAIAVFMVINPHRRGTDSHGEKVQGT-PMWRLFMDPFIACCS 273
D. melanogaster 222 ALVCLLDGLMLLLVMKPVKEAMKQSKDVQDQViPIWRLLMDPYIAVCA 269
M. musculus 222 ACVCLADGILCLTVLKPFSS--RTRENMPVGT-PIYKLMIDPYIAVVA 266
D. rerio 249 AAVSLFDALLLLAVAKPFSAAARARANLPVGT-PIHRLMLDPYIAVVA 295
H. sapiens 249 AAVSLFDALLLLAVAKPFSAAARARANLPVGT-PIHRLMLDPYIAVVA 295
C
Vesicular Acetylcholine Transporter sequence on C-termini end
Tag insertion site
C. elegans 501 NQAQIPNHAVSFQDSRPQAEFPAG-YDPLNPQW 532
D. melanogaster 546 NPFQQQQQQQQQQQQVQSRGPAApANPFRQGF 578
M. musculus 493 GTFAGQSKSFSEEETSEPEYI------------ 513
D. rerio 515 GPFDGCEDDYNYY-SRS---------------- 530
H. sapiens 516 GPFDACEDDYNYYYTRS---------------- 532
D
unc-17::mKate2 (ot907)
DNC
Nerve ring
VNC
E
UNC-17 predicted topology
untagged = olaEx5703
1 2 3 4 5 6 7 8 9 10 11 12
olaEx5704
olaEx5400
syb7251
ot907
ola503
F
Thrashing Assay
**** NS NS * ** NS NS
**** *** NS * * ****
Thrashes per minute
150
100
50
0
Wild type
unc-17(e245)
unc-17(e245); olaEx5703
unc-17(e245); olaEx5400
unc-17(e245); olaEx5704
ot907
syb7251
ola503

Figure S5

bioRxiv preprint doi: https://doi.org/10.1101/2025.08.18.670838; this version posted August 18, 2025. The copyright holder for this preprint (which was not certified by peer review) is the author/funder, who has granted bioRxiv a license to display the preprint in perpetuity. It is made available under aCC-BY 4.0 International license.

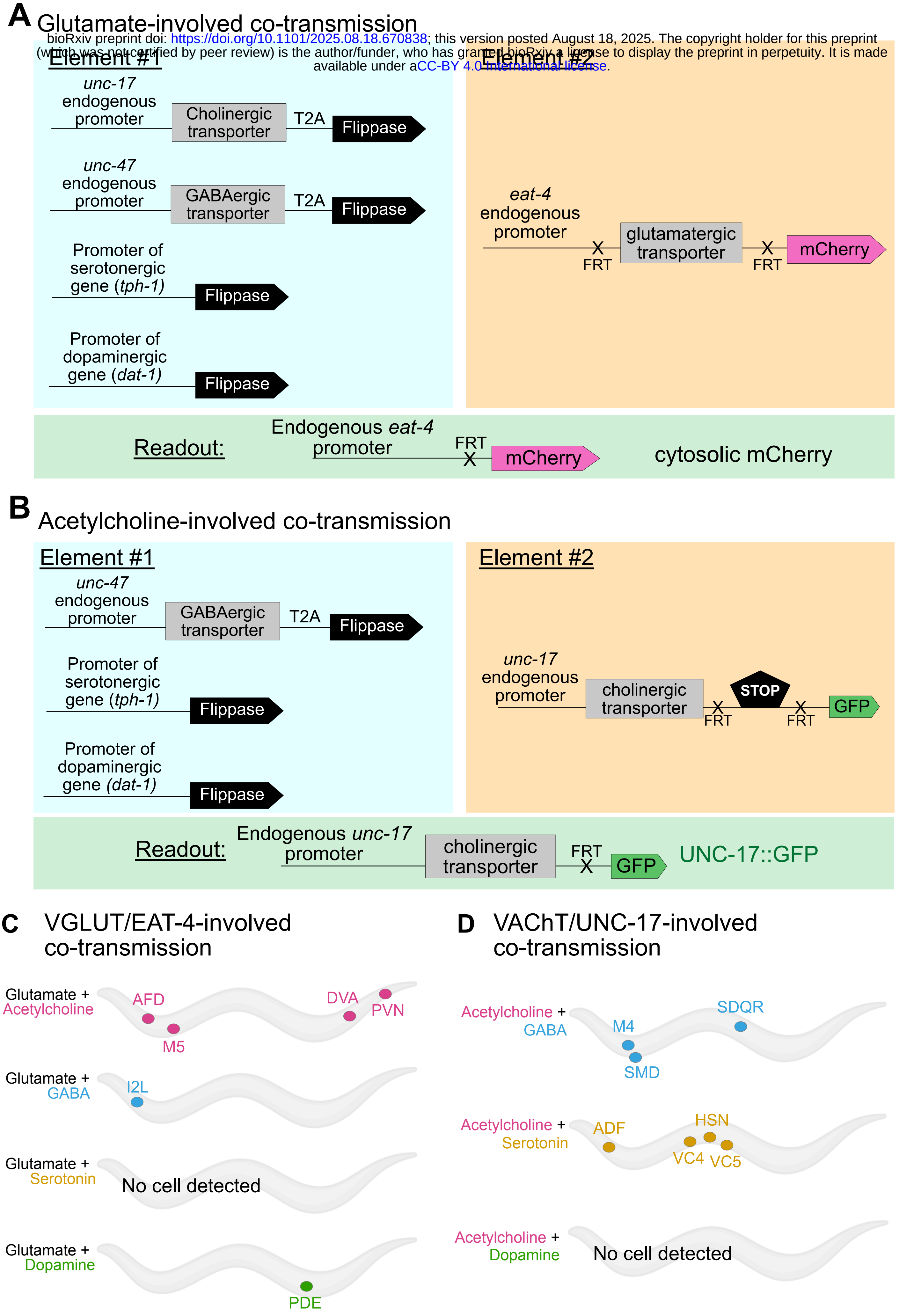

Figure S6

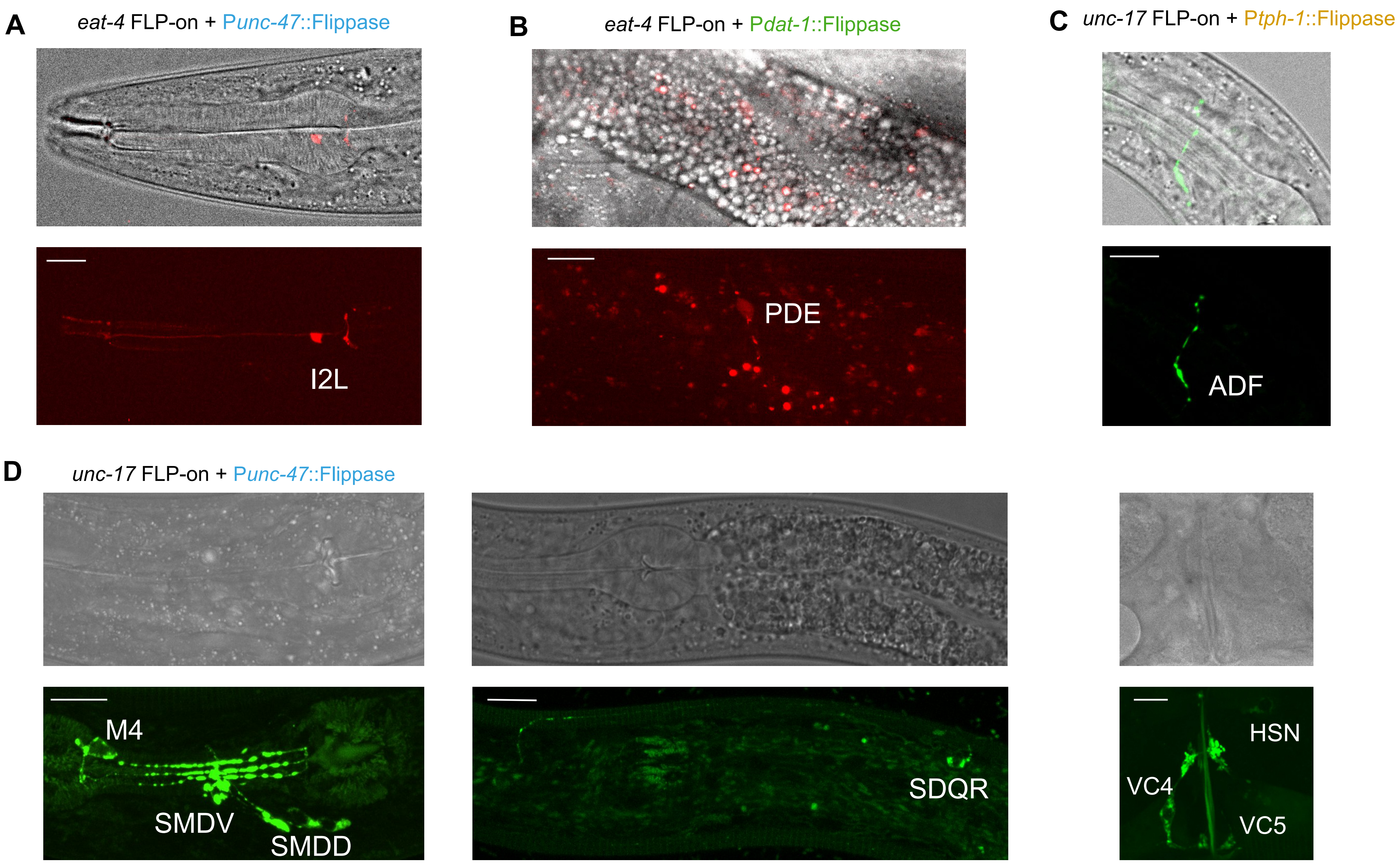
A
eat-4 FLP-on + P*unc-47*::Flippase
I2L
B
eat-4 FLP-on + P*dat-1*::Flippase
PDE
C
unc-17 FLP-on + P*tph-1*::Flippase
ADF
D
unc-17 FLP-on + P*unc-47*::Flippase
M4
SMDV
SMDD
SDQR
HSN
VC4
VC5

Figure S7

bioRxiv preprint doi: https://doi.org/10.1101/2025.08.18.670838; this version posted August 18, 2025. The copyright holder for this preprint (which was not certified by peer review) is the author/funder, who has granted bioRxiv a license to display the preprint in perpetuity. It is made available under aCC-BY 4.0 International license.

Co-transmission atlas of the *C. elegans* nervous system

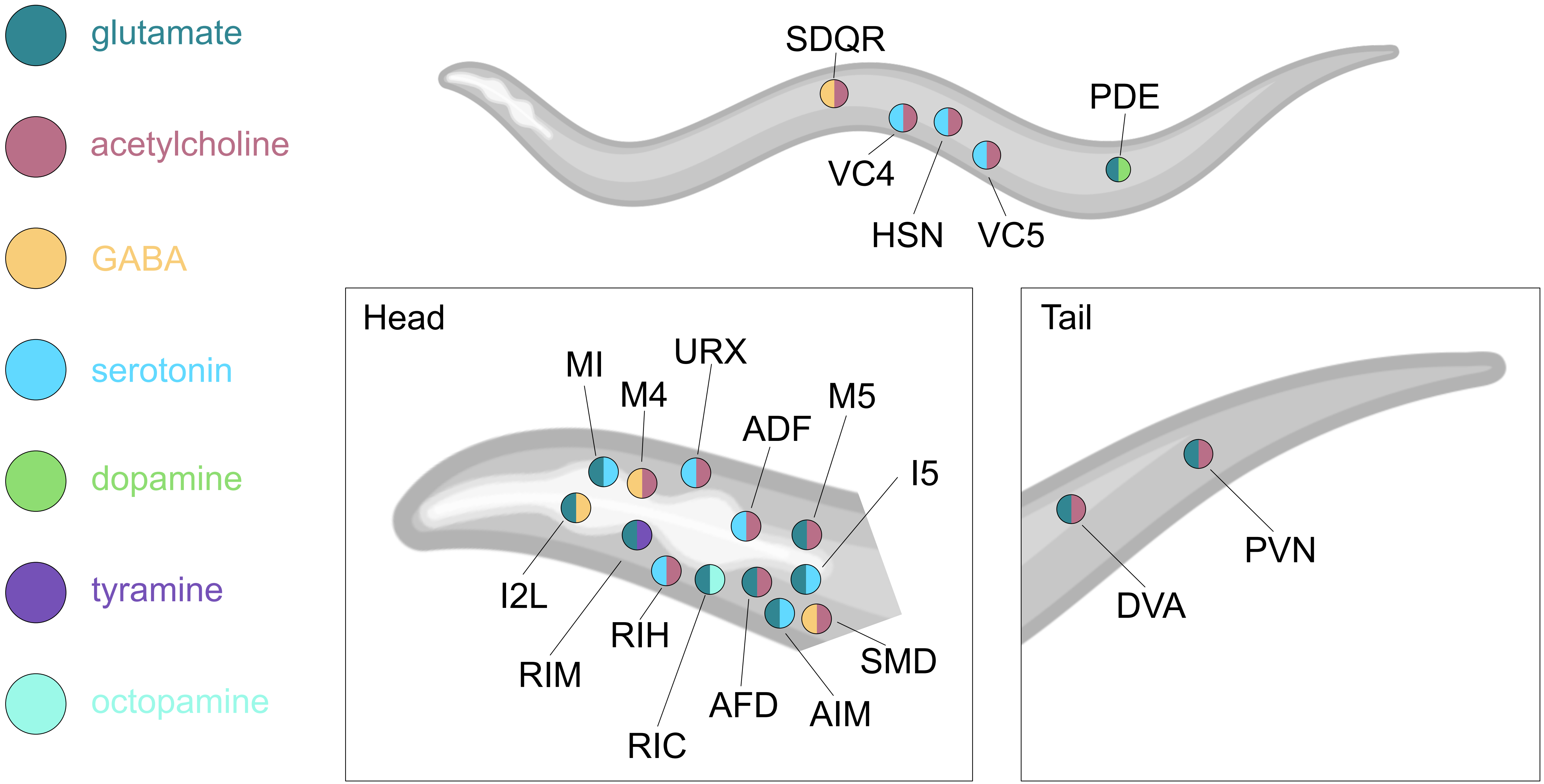

Figure S8

bioRxiv preprint doi: https://doi.org/10.1101/2025.08.18.670838; this version posted August 18, 2025. The copyright holder for this preprint (which was not certified by peer review) is the author/funder, who has granted bioRxiv a license to display the preprint in perpetuity. It is made available under aCC-BY 4.0 International license.

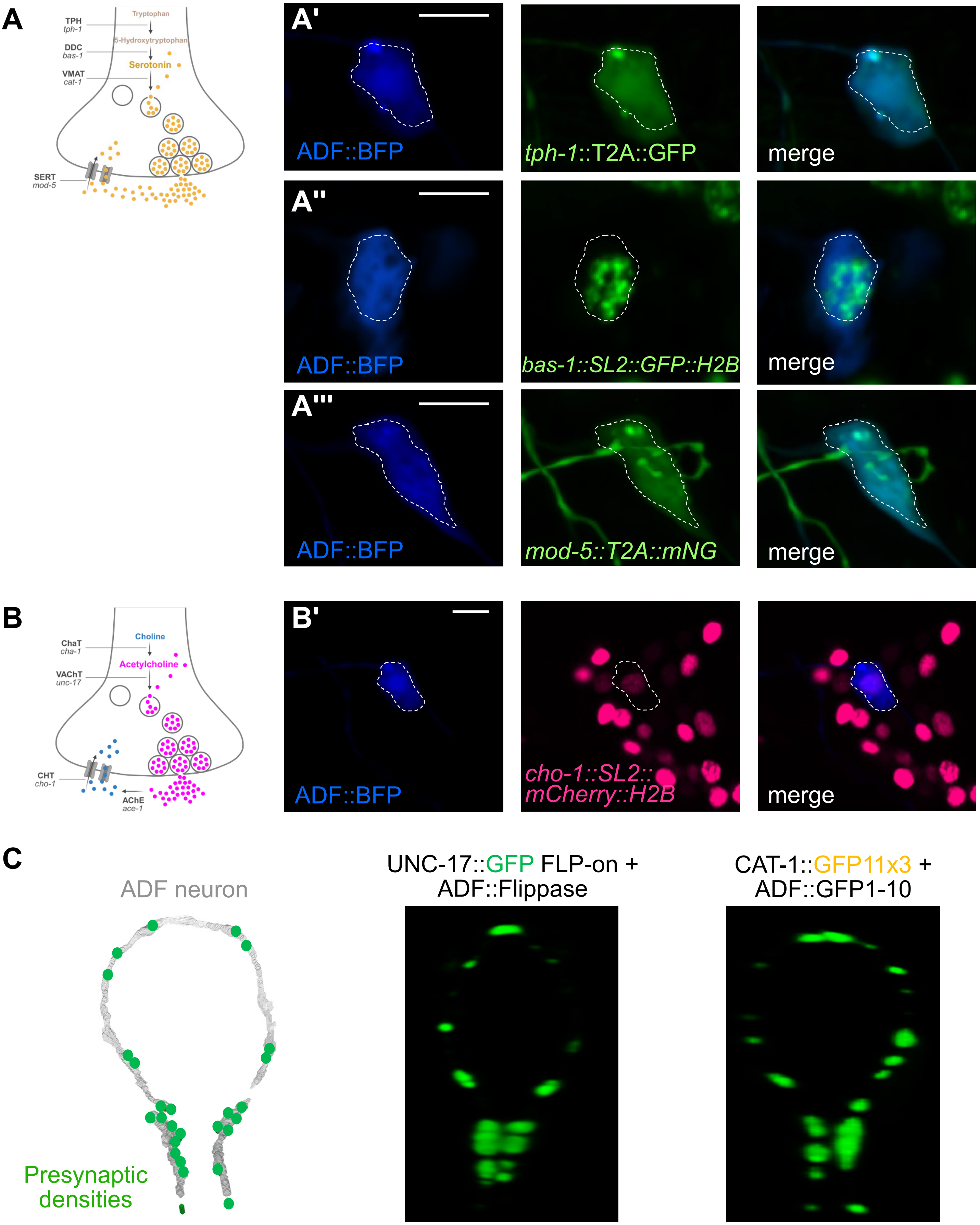

Supplement: Supplement 1 [file NIHPP2025.08.18.670838v1-supplement-1.pdf]
